# Three dimensional evaluation of soft tissue after orthognathic surgery

**DOI:** 10.1186/s13005-018-0179-z

**Published:** 2018-10-05

**Authors:** Junho Jung, Chi-Heun Lee, Jung-Woo Lee, Byung-Joon Choi

**Affiliations:** Department of Oral and Maxillofacial Surgery, School of Dentistry, Kyung Hee University, 26, Kyungheedae-ro, Dongdaemun-gu, 02447 Seoul, Republic of Korea

**Keywords:** Orthognathic surgery, Structured light-based scanners, 3D measurement, Nasolabial soft tissue

## Abstract

**Background:**

To evaluate the nasolabial soft tissue change three-dimensionally after orthognathic surgery, using a structured light scanner.

**Methods:**

Thirty-two malocclusion patients, who underwent orthognathic surgery, were evaluated. CBCT and 3D facial scans were obtained before surgery and 3 months after surgery. The 3D changes in the 26 landmarks, and the relative ratio of the soft tissue movement to the bony movement, were evaluated.

**Results:**

In the Le Fort I advancement patients, the nasal tip moved 17% forward, compared to the maxillary bony movement, but the nasal prominence decreased 15%. The alar width increased 4 mm after the advancement, and the width decreased 4.7 mm after Le Fort I setback. The relative ratio of the soft tissue movement to the bony movement after bilateral sagittal split osteotomy was about 66% at the Li point in the anteroposterior direction, and it was 21% in the Le Fort I advancement and 14% in Le Fort I setback at the Ls point.

**Conclusion:**

Alar cinch suturing may not be sufficient to overcome the effect of the maxilla advancement compressing the nasal complex. Alar width widening was prevented in Le Fort I setback. However, it is uncertain that the alar cinch suturing was solely responsible. The soft tissue around the mandible tends to accompany the bony movement more than the maxillary area. In addition, structured light scanning system proved to be a useful tool to evaluate the nasolabial soft tissue.

## Background

Orthognathic surgery restores not only the occlusal function, but also aesthetics by improving facial harmony. Needless to say, improving soft tissue esthetics is of the ultimate treatment goal. According to skeletal movements from orthognathic surgery, there are consistent nasolabial soft tissue changes. Consequently, various attempts have tried to establish a correlation between the hard and soft tissue changes [1–4].

Conventional two-dimensional (2D) lateral cephalogram is used to predict and evaluate surgical outcomes before and after surgery. Although these methods are convenient and have economic advantages, due to the limitation of its midsagittal projection and the projection angle and distance, the information of soft tissue changes and the accuracy is lacking and unsatisfactory [5]. Moreover, since various soft tissue closure techniques, including the alar cinch suture and the “V-Y” lip mucosal closure, control the nasal base and upper lip [6–8], 2D imaging methods may not be adequate to evaluate the nasolabial tissue.

In recent years, 3D imaging has gained popularity to overcome the limitations of 2D analysis. Cone beam computed tomography (CBCT) provides sufficient information about skeletal structures, and is used to measure soft tissue changes [9]. Moreover, it is frequently applied in dentistry. However, owing to its low resolution and lack of data of skin texture and color, the accuracy of the evaluation for facial soft tissues is not guaranteed [10]. Therefore, noncontact optical scanning methods such as Laser surface scanning and light emitting diode (LED) white light scanning, were introduced as 3D imaging tools [11]. The LED white light scanning has advantages of innocuousness to the human eyes and immediacy to acquire data, making the scanning method suitable for long-term studies and for a larger population of patients [12].

This study was undertaken to assess and describe the nasolabial soft tissue changes three-dimensionally, after bilateral sagittal split osteotomy (BSSRO) or Le Fort I osteotomy with BSSRO, using structured light system- one of the LED white light scanning system. In addition, this study also investigated the effect of the alar base cinch suture following Le Fort I osteotomy, by measuring nasal soft tissue.

## Methods

### Subjects

The patients enrolled for this study included 32 malocclusion cases (17 men, 15 women; mean age, 23.8 ± 3.60 years; range, 17–33 years) who had undergone BSSRO or/and Le Fort I advancement or setback osteotomy, between 2010 and 2016, in the department of oral and maxillofacial surgery at Kyung Hee university Medical Center (Table 1). The patients were divided into 3 groups: BSSRO only (9 patients; mean age, 23.2 ± 3.5; range, 19–31), Le Fort I advancement (13 patients; mean age, 24.0 ± 3.4; range, 17–31), and Le Fort I setback (10 patients; mean age, 24.1 ± 4.1; range, 19–33). All subjects underwent pre- and post-operative orthodontic treatment. In each case, the surgical procedure consisted of BSSRO only, or Le Fort I osteotomy with BSSRO and rigid fixation. After Le Fort I osteotomy and rigid fixation with four L-type titanium plates, alar cinch suture was applied to prevent widening of the nose for all patients. A hole was made at the anterior nasal spine through lateral to lateral by a 1.6 Ø drill. The suture (2/0 non-absorbable (Ethibond)) was passed through the perinasal musculature and fibroadipose tissue at the right alar base from a lateral to medial, the needle then passed through the hole in the nasal spine. The tissue at the left alar base was grasped by the same suture, and the needle then passed through the hole again. A knot of the suture was made at the alar base. The mandible was set back with BSSRO, and the proximal segment fixed with one 4-hole plate and screws. A surgical wafer was placed for 5–6 weeks postoperatively, and inter-arch elastics were used in order to stabilize the inter-maxillary relationship with the surgical wafer. This study was approved by the Ethics Committee at the Kyung Hee University Dental Hospital (KHD IRB 1603–4).

**Table 1:** Details of the subjects

Group	Mean age	Gender (M/F)	Diagnosis	Ethnicity
Le Fort I Advancement & BSSRO	24.0 ± 3.4	9/4	Class I malocclusion: 1	Korean
			Class II malocclusion: 2	
			Class III malocclusion: 10	
Le Fort I Setback & BSSRO	24.1 ± 4.1	4/6	Class I malocclusion: 3	Korean
			Class II malocclusion: 2	
			Class III malocclusion: 5	
BSSRO	23.2 ± 3.5	4/5	Class I malocclusion: 0	Korean
			Class II malocclusion: 0	
			Class III malocclusion: 9	

Patients who underwent superior or inferior positioning of the maxilla and previous nasal surgery and have any craniofacial anomalies and a history of trauma were excluded in this study.

### Data acquisition

3D facial image scans using a LED white light scanning system (Morpheus 3D, Morpheus Co., Ltd., Seoul, Korea) were acquired preoperatively and at 3 months postoperative (scan time: 0.8 s, 33 frame rate: 15 frames/s, data accuracy: ±0.2 mm). Each patient was instructed to relax their lips and set the head in a natural position, while the images were taken. Afterwards, three images from three different views were reconstructed into one 3D image by a merging process.

CBCT scans were acquired preoperatively and at 3 months postoperative, using the Alphard 3030 Dental CT system (Asahi Roentgen Ind. Co., Ltd., Kyoto, Japan) with the following imaging protocol: 80 kVp, 5 mA, 17 s, and 15.4 cm × 15.4 cm field of view. All patients were seated upright with maximum intercuspation, and the Frankfort horizontal (FH) plane of the patient was maintained parallel to the floor during scanning. Voxels were isotropic, each of 0.3 mm width. The CBCT data were exported into the DICOM format, and OnDemand 3D (CyberMed Inc., Seoul, Korea) was used to measure the movement of the maxilla and mandible by superimposition of the data. The actual movement of B-point was measured after BSSRO; both A-point and B-point were measured after Le Fort I osteotomy.

### Landmarks and coordinate system

**Fig. 1:**
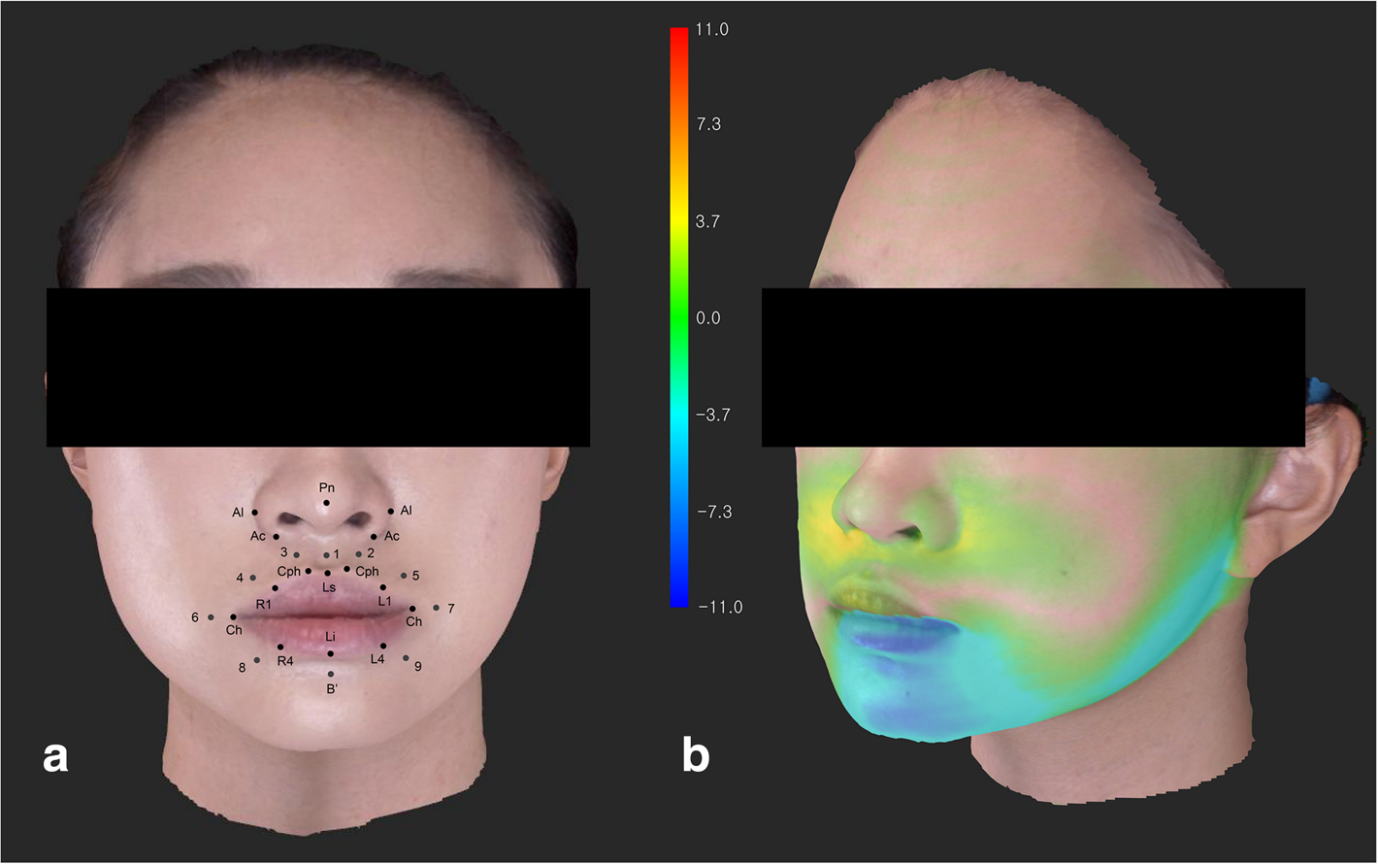
(**a**) Three-dimensionally reconstructed facial image and twenty-six landmarks around the nasolabial tissue. (**b**) A superimposed color map image of facial soft tissue change after orthognathic surgery

**Fig. 2:**
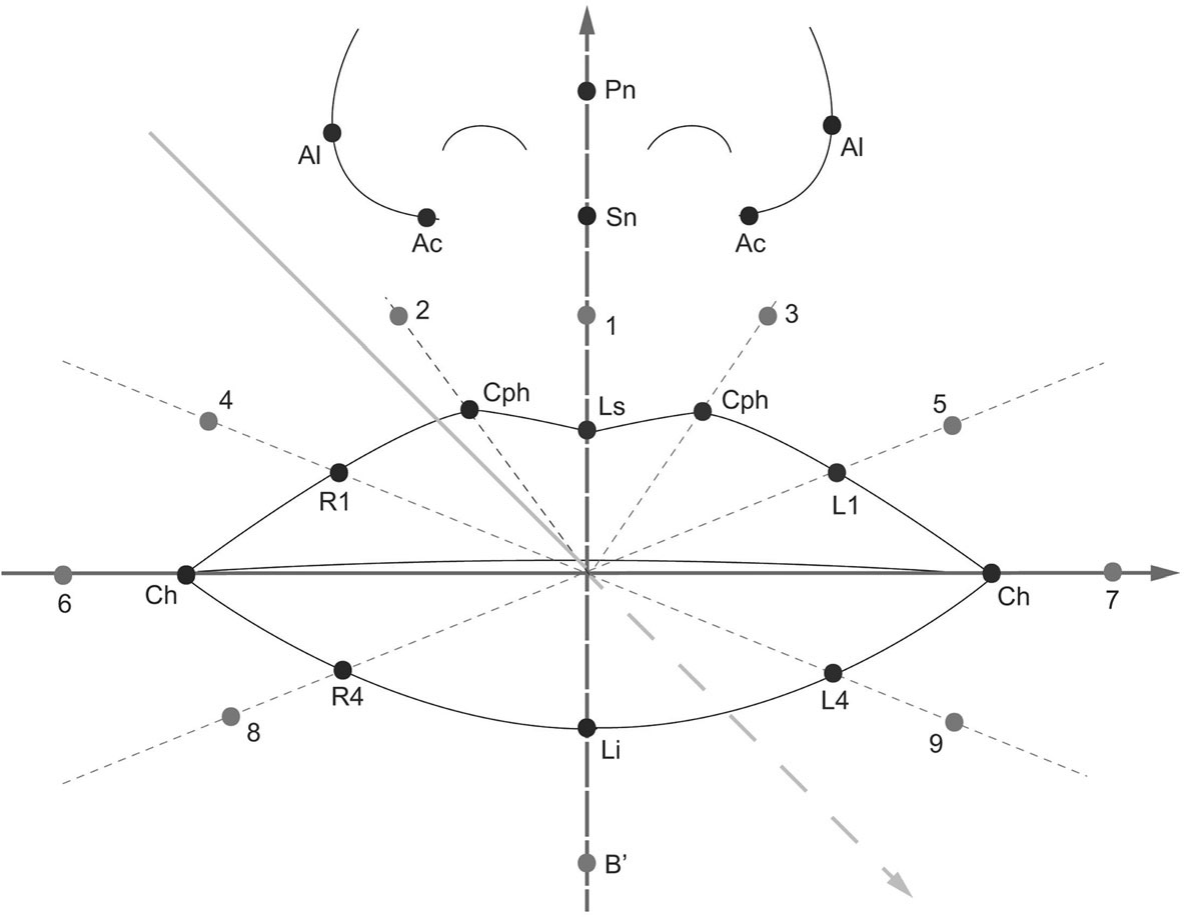
Twenty-six landmarks around the nasolabial tissue [12]

To evaluate the nasolabial soft tissue changes after surgery, 26 landmarks were set on the 3D image along the lip border, and around the lip and the nostrils (Figs. 1a, 2, Table 2). [12] The landmark values were recorded with a 3D Cartesian coordinate system. The left (*x*-axis), front (*z*-axis) and above (*y*-axis) of a patient were defined as positive values; the reference planes were set as below [13]:

**Table 2:** Definitions of the landmarks

Region	Landmarks	Description
Along the lips	Cph (L), Cph (R)	The highest point of the Cupid’s bow (left, right)
	Ls	The lowest point of the middle area of the Cupid’s bow
	Ch (L), Ch(R)	The most lateral point of vermilion border (left, right)
	Li	The lowest point of vermilion border
	L1, L4, R1, R4	Midpoints of curved vermilion border
Around the lips	1–9 B′	Perioral points which are counterparts of each point along the lip border, being apart by the same distance Soft tissue B point
Around the nostril	Al (L), Al (R)	Most lateral point of the alar contour (left, right)
	Pn	Most anterior point of the nose
	Sn	Midpoint of the nasolabial angle at the columellar base
	Ac (L), Ac (R)	Labial insertion of each alar base

The horizontal reference plane (*x*-axis): the ala-tragus plane was rotated 7.5 degree upward on the axis connecting both tragi, and translated until soft tissue nasion.

The sagittal reference plane (*y*-axis): the plane perpendicular to the horizontal plane and passing the soft tissue nasion.

The coronal reference plane (*z*-axis): the plane perpendicular to the other reference planes and passing the soft tissue nasion.

Superimposition of pre-and post-operative image data was performed by reference points including left and right exocanthion, endocanthion, N′, and wide face of forehead using 3D Image Overlay [13]. The differences were evaluated by superimposed 3D image with one coordinate system.

### Statistical analysis

Two weeks after the first measurement, re-evaluation was performed by the same investigator, and both values were compared using the Pearson correlation analysis. The coefficient was above 0.98 at the 95% confidence level. Thus, the mean of the two values was used for the statistical analysis. The Shapiro-Wilk test was used to confirm the Normality of the data distribution. The paired Student *t-*test analyzed the data, and α-level was set to 0.05.

## Results

**Fig. 3:**
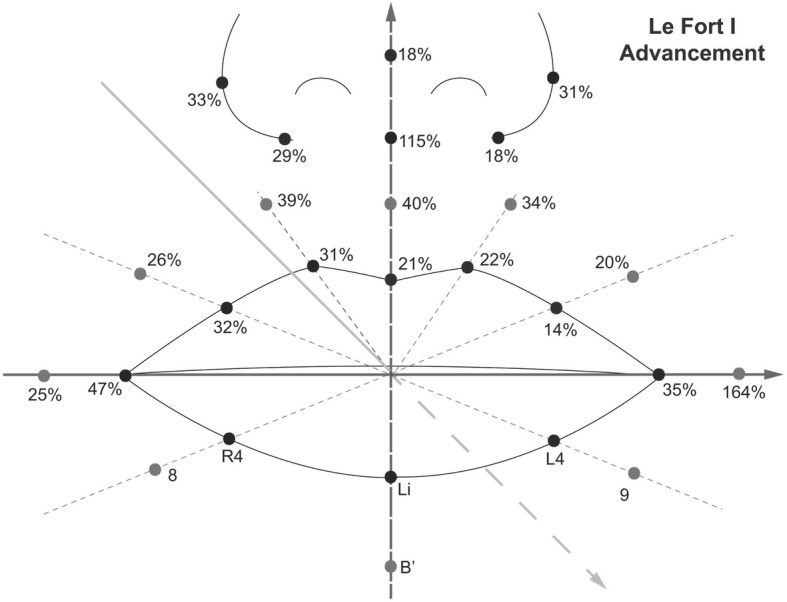
The relative ratio of the soft tissue movement to the bony movement in the anteroposterior direction after Le Fort I advancement osteotomy (%)

The amount of movement at point A was about 2.4 mm (±1.4) after the Le Fort I advancement, and the relative ratio of the soft tissue movement to the bony movement are shown in Table 3 and Fig. 3. Unplanned superoinferior movements of the maxilla was not observed during the measurement. The alar width increased approximately 4 mm after surgery, which was statistically significant. In addition, a significant forward movement of almost all upper lip landmarks was observed due to the maxilla advancement (*P* < 0.05). In the upper lip, the percentage of the soft tissue movement compared to the bony movement was 14–31%. In the nasal area, the ratio was 18–48%, which was higher than the lip area. However, the nasal tip movement was the least among the nasal areas.

**Table 3:** The relative ratio of soft tissue movement to bony movement in the anteroposterior direction and three-dimensional changes of perioral soft tissue after Le Fort I advancement & BSSRO

Landmarks	Soft tissue/bony movement (%)	X-axis			Y-axis			Z-axis		
		mean	SD	*P*-value	Mean	SD	*P*-value	Mean	SD	*P*-value
Cph (L)	22%	−0.21	0.54	0.19	0.36	1.54	0.42	0.53	1.72	0.07
Cph (R)	31%	0.19	0.54	0.24	0.29	1.09	0.35	0.73	1.52	^*^0.02
Ls	21%	−0.07	0.17	0.16	2.85	8.55	0.25	0.51	1.87	^*^0.00
Lip (L1)	14%	0.11	1.16	0.73	0.46	1.39	0.26	0.34	1.80	^*^0.00
Lip (R1)	32%	−0.01	0.91	0.98	1.01	2.57	0.19	0.77	1.53	^*^0.00
Ch (L)	35%	0.63	1.05	0.05	0.94	1.88	0.10	−1.72	3.17	0.29
Ch (R)	47%	1.24	5.77	0.45	0.80	2.88	0.34	−2.35	3.20	0.11
1	40%	0.04	0.25	0.57	−0.11	0.87	0.67	−0.96	1.46	^*^0.04
2	39%	0.29	0.61	0.11	0.31	0.55	0.07	−0.93	37.24	0.39
3	34%	0.39	0.51	^*^0.02	−0.09	0.88	0.71	−0.82	1.44	0.62
4	26%	0.18	0.82	0.45	−0.02	0.88	0.95	−0.63	1.61	0.18
5	20%	0.08	0.83	0.74	0.14	0.93	0.59	−0.49	1.87	0.37
Al (L)	31%	1.23	0.80	^*^0.00	0.02	0.36	0.88	0.74	0.09	^*^0.01
Al (R)	33%	2.89	5.51	^*^0.04	0.14	0.47	0.29	0.78	1.06	^*^0.02
Ac (L)	18%	−5.17	13.75	0.20	−1.87	12.03	0.59	0.44	7.85	0.84
Ac (R)	29%	1.66	10.43	0.13	−3.82	15.25	0.38	0.70	5.70	0.67
Sn	115%	0.04	1.72	0.93	−2.05	11.91	0.52	−2.74	4.03	^*^0.03
Pn	18%	−0.04	0.37	0.72	0.02	0.22	0.71	0.43	0.43	^*^0.00

*SD* standard deviation

Asterisks indicate statistical significance between the values of pre- and post-operative measurement (*P* < 0.05)s

**Fig. 4:**
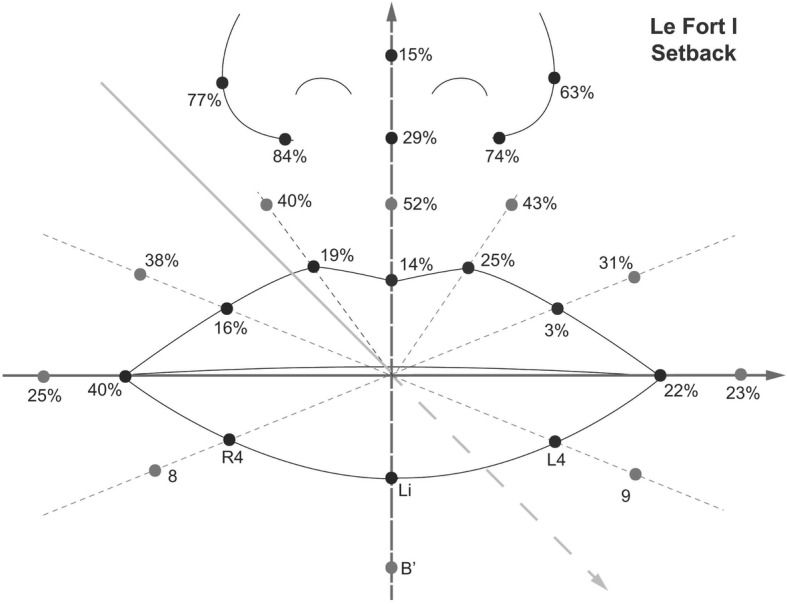
The relative ratio of the soft tissue movement to the bony movement in the anteroposterior direction after Le Fort I setback osteotomy (%)

After the Le Fort I setback osteotomy, on an average, point A moved posteriorly by about 2.1 mm (±1.0). Unplanned superoinferior movements of the maxilla was not observed during the measurement. Also, postoperatively the alar width decreased about 4.7 mm (Table 4, Fig. 4). In the upper lip area, the soft tissue movement was 3–52% compared to the bony movement, and it was 15% at the nasal tip. However, the proportion was 63–84% at the alar and alar base areas.

**Table 4:** The relative ratio of soft tissue movement to bony movement in the anteroposterior direction and three-dimensional changes of perioral soft tissue after Le Fort I setback & BSSRO

Landmarks	Soft tissue/bony movement (%)	X-axis			Y-axis			Z-axis		
		mean	SD	*P*-value	Mean	SD	*P*-value	Mean	SD	*P*-value
Cph (L)	25%	− 0.32	0.56	0.13	0.39	1.26	0.38	−0.52	0.92	0.13
Cph (R)	19%	0.33	0.44	0.06	7.23	20.66	0.32	−0.41	0.93	0.23
Ls	14%	0.09	0.41	0.52	0.27	1.29	0.55	−0.29	0.95	0.39
Lip (L1)	3%	−0.06	0.38	0.65	0.26	0.85	0.38	−0.06	0.88	0.84
Lip (R1)	16%	−0.54	1.74	0.38	0.21	0.64	0.36	−0.33	0.62	0.15
Ch (L)	22%	0.02	0.62	0.94	0.48	1.45	0.35	0.96	2.72	0.32
Ch (R)	40%	−0.52	1.02	0.17	−7.08	21.63	0.36	1.69	3.13	0.14
1	52%	0.02	0.10	0.51	−0.45	0.39	^*^0.01	−1.10	0.65	^*^0.00
2	40%	0.37	0.37	^*^0.02	−6.79	18.91	0.31	−0.83	0.65	^*^0.01
3	43%	−0.49	0.38	^*^0.01	−0.51	0.58	^*^0.03	−0.90	0.72	^*^0.01
4	38%	0.43	0.58	0.06	−0.19	0.41	0.19	−0.80	1.01	^*^0.04
5	31%	−0.28	0.40	0.07	−0.12	0.15	^*^0.04	−0.64	0.73	^*^0.03
Al (L)	63%	−1.26	1.29	^*^0.01	−1.92	5.47	0.32	−1.32	0.93	^*^0.00
Al (R)	77%	−3.42	13.50	0.47	−0.06	0.11	0.15	−1.62	2.22	0.06
Ac (L)	74%	−1.26	1.29	^*^0.01	−1.92	5.47	0.32	−1.32	0.93	^*^0.00
Ac (R)	84%	−3.42	13.50	0.47	−0.06	0.11	0.15	−1.62	2.22	0.06
Sn	29%	−0.09	0.12	0.06	−2.72	9.20	0.40	−0.62	1.31	0.20
Pn	15%	−0.03	0.05	0.13	0.01	0.04	0.69	−0.32	0.41	^*^0.04

*SD* standard deviation

Asterisks indicate statistical significance between the values of pre- and post-operative measurement (*P* < 0.05)

The amount of setback movement after BSSRO was measured as the mean value of 6.6 mm (±2.8) at point B by CBCT (Table 5). The Li and soft tissue B point movement was 4.3 and 4.85 mm, respectively. The relative ratio of soft tissue movement to bony movement was about 66% and 73% in the anteroposterior direction at the landmarks.

**Table 5:** The relative ratio of soft tissue movement to bony movement in the anteroposterior direction and three-dimensional changes of perioral soft tissue after BSSRO

Landmarks	Soft tissue/bony movement (%)	X-axis			Y-axis			Z-axis		
		mean	SD	*P*-value	Mean	SD	*P*-value	Mean	SD	*P*-value
Ch (L)	37%	0.45	0.60	0.09	0.26	1.27	0.60	− 0.18	0.88	0.60
Ch (R)	25%	−0.04	1.19	0.94	−0.33	0.54	0.16	−0.62	0.91	0.13
L4	57%	1.16	1.71	0.12	−13.89	3.94	0.39	−3.77	1.67	^*^0.00
R4	52%	3.32	11.19	0.46	11.60	3.14	0.37	−3.44	1.05	^*^0.00
Li	66%	−0.26	0.82	0.44	11.48	3.11	0.37	−4.35	1.25	^*^0.03
6	114%	−1.17	1.64	0.11	1.08	1.07	^*^0.04	−7.50	2.14	0.39
7	11%	0.51	1.68	0.45	0.82	2.06	0.44	0.73	0.74	^*^0.04
8	55%	−1.26	2.15	0.17	16.70	45.04	0.37	3.61	1.30	^*^0.00
9	309%	1.08	1.47	0.10	−0.12	1.36	0.82	20.39	44.57	0.27
B′	73%	− 0.54	0.69	0.08	−0.91	1.33	0.12	4.82	2.13	^*^0.00

*SD* standard deviation

Asterisks indicate statistical significance between the values of pre- and post-operative measurement (*P* < 0.05)

## Discussion

Le Fort I osteotomy has the potential to alter the mid-facial region. However, despite precise planning and surgery, it is difficult to predict soft tissue changes. Hence, this study aimed to demonstrate the 3D change in postoperative nasolabial soft tissue, in order to optimize surgical outcomes and patient satisfaction. Especially, undesirable effects such as widening of the alar base or alteration of the nasal tip projection [3, 14–16] were measured.

2D techniques have been broadly used until recent years. However, due to their limitations, the measurements might be somewhat biased [14]. Therefore, various methods, including laser scanners, stereophotogrammetry, and structured light systems, have recently been introduced for 3D soft tissue assessment. The accuracy of the structured light system used in this study compares the direct anthropometric measurements, and 3D facial scan values were less than 1 mm [17, 18]. Moreover, as the face of the subject was simultaneously scanned from multiple angles in less than 1 s, we expect more reliable collections of 3D datasets. The facial scanning device used in study is now available more than 10 countries in America, Europe and Asia. The cost for the procedure is less than the one for CBCT taking in our clinic.

In our study, the nasolabial soft tissue changes were compared with the actual movement of point A measured in CBCT. The average movement of the nasal tip was about 17% compared to the maxillary bony movement. However, the nasal prominence (measured from the alar groove to the tip) decreased by about 15% of the total maxillary advancement. In other words, although the actual nasal tip moved forward, the nasal prominence itself decreased as the piriform aperture compressed the nasal complex, and it widened and became obtuse. According to the tripod theory, the widened alar may pull the nasal tip back, despite an increase in relative nasal tip position to the face, so that intrinsic nasal tip projection decreased while the extrinsic nasal projection increased [19–21].

In this study, despite alar cinch suturing, the alar width resulted in an increase of 4 mm after Le Fort I advancement. However, in case of Le Fort I setback, the width decreased by about 4.7 mm. Therefore, we surmise that the effect of the maxilla advancement compressing the nasal complex exceeds the effect of the cinch suturing. Although alar width widening was prevented in Le Fort I setback, it is uncertain that the alar cinch suturing was solely responsible, because the backward movement of the supporting bony structure may also have effects to prevent the widening.

The relative ratio of the soft tissue movement to the bony movement after BSSRO was only about 66% at the Li point, and 73% at B′ point. In Le Fort I advancement, the ratio was 21% at the Ls point, and 14% in the Le Fort I setback. These results indicate that considering the labial tissue, more soft tissue movements occur around the mandible than the maxillary area. A similar tendency was observed in other studies. Biak and Kim reported that the proportion of soft tissue changes to hard tissue changes at the Ls point was 34% in Le Fort I advancement, and 81% at Li point in BSSRO [22]. Soncul and Bamber also presented 49% of the proportion at Upper vermillion in Le Fort I advancement, and 71% at the Lower vermillion in BSSRO [23].

Previous studies describe that nasolabial soft tissue change following Le Fort I osteotomy occurs inconsistently, indicating that hard-to-soft tissue prediction is a difficult task. Nasal tip movement has been reported in 30–60% of the total maxillary advancement, and the columellar base was known to be advanced more than the nasal tip, after the anterior movement of the maxilla [24–26]. However, reports of this finding are also inconsistent [6]. The alar width increased along the maxillary advancement, but with wide ranges. [27–29] Van Loon et al. presented no difference in the alar width after Le Fort I advancement, between patients who had underwent an alar cinch and those who had not [30]. Howley et al. also compared a group of patients who underwent an alar cinch suture with a control group, and concluded that the alar base width eventually increased 6 months after the surgery [31]. These results are in accordance with this study, which implicates that it is hard to control the alar width despite the alar cinch suture after the maxillary advancement osteotomy.

We observed a wide variety of soft tissue response to the bony movement in this study, and this result could originate due to soft adaptation process after surgery. Although this study helps us predict the outcomes of orthognathic surgery, a large sample number is required to increase reliability and confirm the factors that influence the nasolabial soft tissue changes after orthognathic surgery. However, a structured light system such as a 3D imaging method to measure facial soft tissues, provides useful and accurate information conveniently. Hence, this system can serve as a worthwhile tool in dentistry. In addition, there are other factors to observe soft tissue response after orthognathic surgery, such as lip thickness, volume and tonicity, which were not evaluated in this study. It should be evaluated for a future study to better understand soft tissue response.

## Conclusions

Alar cinch suturing does not appear sufficient to overcome the effect of the maxilla advancement compressing the nasal complex. The nasal complex became obtuse and the alar width increased. Alar width widening was prevented in Le Fort I setback. However, it is uncertain that the alar cinch suturing was solely responsible. The soft tissue around the mandible tends to accompany the bony movement more than the maxillary area. In addition, structured light scanning system proved to be a useful tool to evaluate the nasolabial soft tissue.

## Abbreviations

2D: Two-dimensional
3D: Three dimensional
BSSRO: Bilateral sagittal split osteotomy
CBCT: Cone beam computed tomography
LED: Light emitting diode
